# Aflatoxin M_1_ Enrichment in Buffalo Mozzarella and Ricotta: Case Study on Naturally Contaminated Milk and Exploratory Analysis of Sterigmatocystin and Aflatoxicol

**DOI:** 10.3390/molecules31183316

**Published:** 2026-09-18

**Authors:** Maurizio Cossu, Stefano Sdogati, Andrea Sanna, Serenella Orsini, Ivan Pecorelli, Tommaso Pacini, Gilberto Giangolini, Roberto Condoleo, Carlo Boselli, Valentina D’Onofrio

**Affiliations:** 1 Istituto Zooprofilattico Sperimentale della Sardegna “G.Pegreffi”, Via Vienna, n.2, 07100 Sassari, Italy; maurizio.cossu@izs-sardegna.it (M.C.); andrea.sanna@izs-sardegna.it (A.S.); 2 Istituto Zooprofilattico Sperimentale dell’Umbria e delle Marche, Via G. Salvemini, 1, 06126 Perugia, Italy; s.orsini@izsum.it (S.O.); i.pecorelli@izsum.it (I.P.); t.pacini@izsum.it (T.P.); 3 Istituto Zooprofilattico Sperimentale del Lazio e della Toscana “M. Aleandri”, National Reference Centre for Ovine and Caprine Milk and Dairy Products Quality (C.Re.L.D.O.C.), Via Appia Nuova, 141, 00178 Roma, Italy; gilberto.giangolini@izslt.it (G.G.); roberto.condoleo@izslt.it (R.C.); carlo.boselli@izslt.it (C.B.); valentina.donofrio@izslt.it (V.D.)

**Keywords:** enrichment factor, buffalo milk, cheesemaking, aflatoxin M_1_, aflatoxicol, sterigmatocystin

## Abstract

Mycotoxins, including sterigmatocystin (STC), aflatoxicol (AFL), aflatoxin B_1_ (AFB_1_), and its metabolite aflatoxin M_1_ (AFM_1_), represent a major food safety concern in the dairy industry due to their high toxicity and thermal stability. While AFM_1_ in milk is strictly regulated, there is a substantial lack of specific guidelines and scientific data regarding its behavior and enrichment factors (EFs) in buffalo-derived products. Furthermore, knowledge gaps persist regarding the transfer and stability of co-occurring molecules like STC and AFL. This study evaluated the distribution and fate of AFM_1_, STC, and AFL during traditional, laboratory-scale cheesemaking of buffalo mozzarella and ricotta. Fourteen trials were conducted using bulk tank milk naturally contaminated with AFM_1_, alongside an exploratory trial utilizing toxin-free milk artificially spiked with all three targets. Mass balance and specific EFs were calculated across all processing streams. AFM_1_ was detected in all raw milk samples, showing a strong affinity for caseins. Approximately half of the initial milk AFM_1_ content was retained in mozzarella (50.3 ± 3.3%), whereas ricotta retained only a minor fraction (6.9 ± 0.7%), with the majority lost in the second whey (30.7 ± 1.3%). The mean EFs were 2.3 ± 0.3 for mozzarella and 1.1 ± 0.2 for ricotta. AFL was never detected in milk samples while STC was mainly present at concentrations < 1 ng/kg. In the spiked trial, AFL demonstrated stability with an EF of 1.5 in mozzarella, while STC displayed potential degradation within the milk matrix and during sample storage. The experimental EFs determined for buffalo mozzarella and ricotta are lower than the standard factors currently applied by Italian regulations for non-bovine dairy products. These accurate, matrix-specific estimations are essential to prevent the under-reporting of non-compliant samples and to refine international consumer risk assessment models.

## 1. Introduction

Mycotoxins are toxic secondary metabolites produced by filamentous fungi belonging to the genera *Aspergillus*, *Penicillium*, and *Fusarium*, which can contaminate feed and food along the agro-food chain. Among these, aflatoxin B_1_ (AFB_1_) is particularly important due to its high toxicity and carcinogenicity. AFB_1_ is classified by the International Agency for Research on Cancer (IARC) as a Group 1 (carcinogenic to humans). When lactating animals consume feed contaminated with AFB_1_, the toxin is primarily metabolized in the liver to aflatoxin M_1_ (AFM_1_), which is subsequently excreted in milk, urine, and feces. AFM_1_ is thermally stable and resistant to conventional heat treatments, allowing it to persist throughout dairy processing and to be efficiently transferred into milk-derived products such as cheese and ricotta [1]. The mechanism governing this transfer has been extensively investigated: AFM_1_ exhibits a strong affinity (hydrophobic interactions) for casein micelles, leading to its preferential accumulation in curd-based cheeses [2]. A smaller fraction associates with whey proteins and may be retained in ricotta because of whey protein co-precipitation during the thermo-acidic processing.

AFM_1_ is a Group 1 carcinogen as well as AFB_1_ and its presence in milk is regulated within the European Union under Commission Regulation (EU) 2023/915, which sets a maximum permitted level of 0.050 µg/kg [3]. No specific limits are currently established for cheese or whey products. However, Italy applies enrichment factors (EFs) to assess compliance based on cheese texture and ripening, as defined by the Moisture content on a Fat-Free Basis parameter (MFFB), which measures the percentage of moisture relative only to the non-fat solid matter to classify cheese hardness (e.g., soft, semi-hard, or hard).

The Italian Ministry of Health has set specific EFs for cheeses from bovine milk: EF = 3 for soft cheeses (MFFB ≥ 68%), EF = 4 for semi-soft cheeses (62% ≤ MFFB < 68%), EF = 5 for semi-hard (55% ≤ MFFB < 62%) and hard cheeses (47% ≤ MFFB < 55%), and EF = 6 for extra-hard cheeses (MFFB < 47%) [4]. For products derived from non-bovine milk and whey, an EF of 3 is applied to soft cheeses and whey products, whereas an EF of 5.5 is used for hard cheeses. These EFs are utilized to indirectly determine compliance with the regulatory limits set for milk by dividing the measured concentration in dairy products by the corresponding factor.

Sterigmatocystin (STC), a biosynthetic precursor of AFB_1_ mainly produced by xerophilic *Aspergillus* species, is classified as possibly carcinogenic to humans (IARC Group 2B). According to the European Food Safety Authority (EFSA), the in vivo carcinogenic potency of STC is estimated to be approximately three orders of magnitude lower than that of AFB_1_. STC has been detected in cereals and mold-contaminated cheeses [5].

In 2013, the European Food Safety Authority (EFSA) highlighted the scarcity of data available to reliably assess the transfer rate of STC from contaminated feed into ruminant milk. Although STC-producing fungi are widespread in the environment, documented cases of STC contamination in milk and dairy products have been relatively rare. Nonetheless, more recent studies have successfully detected STC residues in both milk and commercial cheeses [6].

Despite these findings, current data remain inadequate to conclusively determine the extent to which STC is transferred from feed to milk and, subsequently, to dairy products. Aflatoxicol (AFL) is a reduced hydroxylated metabolite of AFB_1_, with an estimated genotoxic potency of approximately 70% compared to the parent compound. AFL can be conjugated with glucuronic acid and excreted via urine and milk, or reconverted to AFB_1_ by hepatic enzymes, contributing to a reversible metabolic cycle that may prolong its toxic effects [7]. Although its higher water solubility suggests a potentially enhanced elimination, this reversible interconversion limits effective detoxification. According to the European Food Safety Authority [8], in vitro studies using human liver cells indicate that AFL exhibits genotoxic potency lower than that of AFB_1_ but higher than AFM_1_. Due to its higher water solubility, AFL is presumed to exhibit lower affinity for casein micelles, potentially leading to greater retention in whey fractions. However, further investigation is needed to elucidate the distinct behavior of this mycotoxin during dairy processing. AFL has been detected in cheeses produced from cow’s milk, with some studies reporting concentrations exceeding those of AFM_1_, while others have observed lower levels. Currently, no legal limits exist for STC and AFL; however, EFSA has expressed interest in monitoring these mycotoxins due to their potential health risks. Recently, the Italian Ministry of Health has recommended further investigations into the presence of AFM_1_, AFL, and STC in cheese to address significant knowledge gaps [4].

Concerning STC, current evidence on its occurrence in milk and dairy products is insufficient to support a comprehensive risk assessment. Available data primarily suggest direct contamination during the ripening process, as a result of *Aspergillus* spp. proliferation on the cheese rind. Regarding AFL, although data remain scarce, its presence in milk and cheese has been reported [9]. Considering AFL’s toxicity, potential genotoxicity, and its ability to be reconverted to AFB_1_ within the body, further research is imperative to elucidate its occurrence in dairy products and assess consumer exposure, including biomonitoring approaches.

In addition to previous considerations, it is important to note that the majority of existing studies have focused on cow milk, with relatively scarce data available on non-bovine dairy species. This gap is noteworthy, as the transfer dynamics of mycotoxins from contaminated feed to milk may differ significantly between buffaloes and cattle. Furthermore, distribution and partitioning of these toxins during dairy processing could vary in buffalo-derived products, such as mozzarella and ricotta, which are not only widely consumed but also have considerable cultural and economic importance. Italy is a leading global producer and exporter of buffalo dairy products, encompassing both renowned Protected Designation of Origin (PDO) commodities such as Mozzarella di Bufala Campana and Ricotta di Bufala Campana and their non-PDO counterparts, all of which maintain a strong presence in international markets.

To address these gaps, the present study aimed to quantify the distribution and fate of AFM_1_ across all stages of buffalo dairy processing—including intermediate fractions, final products, and by-products—utilizing a comprehensive mass balance analysis. Establishing matrix-specific enrichment factors (EFs) for both mozzarella and ricotta cheeses based on traditional manufacturing practices.

Regarding AFL and STC, the investigation evaluates their potential co-occurrence in buffalo milk naturally contaminated with AFM_1_, alongside an exploratory study for the assessment of their stability and partitioning throughout the cheesemaking process.

## 2. Results and Discussion

### 2.1. Milk Composition and Cheese Yield Performance in Buffalo Mozzarella and Ricotta

The chemical composition of milk is a critical determinant of the yield and quality of both ricotta and mozzarella. Protein and fat content play a central role in achieving satisfactory mozzarella yields by increasing curd mass and affecting the final texture. In contrast, ricotta yield is primarily influenced by the concentration of soluble proteins, such as whey proteins, that remain in the whey after curd separation.

Buffalo milk typically provides higher yields of both mozzarella and ricotta than cow milk due to its naturally higher concentrations of fat and protein. On average, buffalo milk contains 4.26–7.16% fat and 4.12–5.62% protein, whereas cow milk generally ranges from 3.3–5.4% fat and 3.0–3.9% protein. These values may vary depending on breed, diet, and stage of lactation [10,11].

In the milk samples used in the present study, protein content ranged from 4.0% to 5.1%, while fat content varied from 4.0% to 8.2%. In mozzarella, protein content ranged from 12% to 26% and fat from 11% to 32%. In ricotta, protein content ranged from 8.1% to 17%, whereas fat ranged from 11% to 22%. Chemical composition of buffalo milk used in the cheesemaking trials is detailed in Supplementary Material (Table S1).

When considering only the initial milk composition, protein content showed relatively limited variability across batches, whereas fat content exhibited a more pronounced variation, particularly in batches 3 and 4, which displayed generally lower values. As expected, this variability was reflected in the final products, especially in terms of reduced fat content in mozzarella. Excluding these two batches, the remaining samples showed more homogeneous and comparable compositional profiles.

Yield was calculated as the weight of the final product, mozzarella or ricotta, divided by the initial milk weight and expressed as a percentage. Mozzarella yields ranged from 17% to 24%. A slightly atypical behavior was noted for batch 7, which showed a yield of 17.3% despite exhibiting a markedly higher fat content compared to the other two low-yield batches. Overall, the data indicate yields in line with average relevant literature, considering also the small-scale size of these experiments (initial milk weight: approximately 4 kg) [12,13]. For ricotta, yields ranged between 3.5% and 8.1%, showing the same trend observed for mozzarella for batches 3 and 4, as well as the anomalous value of batch 7. In this case, as well, the yields were consistent with those reported in controlled studies using buffalo whey. The satisfactory outcome of the cheesemaking process was pivotal for further consideration of mycotoxin fate and EF calculation.

### 2.2. Analytical Results

The analytical methodology was developed as detailed in Cossu et al. 2025 and validated as reported in Sdogati et al. 2026 following Regulation 2782/2023 [14,15,16].

Each sample obtained from the experimental cheesemaking trials was analyzed for the presence of AFM_1_, STC, and AFL. Table 1 reports the concentrations measured in trials conducted with milk naturally contaminated with AFM_1_ (batches 1 to 14–B1 to B14), while Table 2 presents the results obtained from the exploratory trial using spiked milk (spike batch). The data processing and calculation of key statistical descriptors were performed according to the following criteria: when a mycotoxin was detected at levels below the limit of quantification (LOQ), it was reported in the table as “det,” and for statistical purposes, a value equal to half the LOQ was assigned. If the analyte was not detected and was below the limit of detection (LOD), indicated in the table by the symbol “<,” a value of zero was assigned.

As expected, AFM_1_ was detected in all analyzed raw milk samples. AFM_1_ concentrations in milk ranged from 6.0 to 163 ng/kg, with a mean value of 44 ng/kg. Corresponding levels in mozzarella ranged from 15 to 476 ng/kg (mean: 107 ng/kg), while ricotta exhibited concentrations between 4.2 and 192 ng/kg (mean: 52 ng/kg). Data showed that AFM_1_ concentrations in mozzarella were approximately two- to three-fold higher than those found in the corresponding milk samples. Conversely, AFM_1_ levels in ricotta were comparable to those in milk. The data obtained for AFM_1_ from the spike batch showed that the concentration measured in milk was consistent with the expected level, and the distribution across the different cheesemaking products was in line with that observed in naturally contaminated milk, confirming the reproducibility of the distribution pattern. This aspect will be examined in detail in the following sections.

STC was detected in 12 of the 14 naturally contaminated batches, considering at least one positive result among the analyzed matrices for each batch. However, many measurements were close to or below the limit of detection (LOD), limiting the reliability of quantitative assessment and the interpretation of STC distribution across matrices. The highest concentrations observed among all analyzed batches were 1.3 ng/kg in milk (batch 9), 10 ng/kg in mozzarella (batch 4), and 4.1 ng/kg and 4.0 ng/kg in ricotta (batches 3 and 4, respectively).

Notably, marked discrepancies were observed when comparing STC concentrations in milk with those detected in mozzarella and ricotta. In batch 4, STC was undetectable in milk, whereas both mozzarella and ricotta from the same batch exhibited the highest concentrations recorded in the study. Conversely, in batch 3, STC was detected in milk and ricotta but was absent in mozzarella. When AFM_1_ distribution was considered as a reference pattern, batches 2, 9, 10, and 14 exhibited STC trends more closely aligned with the expected partitioning behavior.

Regarding intermediate products and by-products of processing, the majority of samples tested negative for STC. Based on the available data, a possible degradation of STC between the cheesemaking phase and the analytical determination may have occurred in certain batches or specific matrices within the same batch. This hypothesis is supported by the results of the spike batch, in which STC was the only mycotoxin showing a substantial discrepancy between the expected and measured concentration in the fortified starting milk (33 ng/kg detected vs. 50 ng/kg expected).

Regarding AFL, although its occurrence in milk and dairy products has been reported in recent studies, it was not detected in the samples analyzed in the present work. However, the limited number of samples prevents definitive conclusions.

For the fortified samples, the AFL concentration measured in milk was consistent with the expected level, confirming that the mycotoxin remained stable, similar to AFM_1_. Moreover, the data indicates that AFL was transferred into cheesemaking products. Further evaluation of these results will be presented in the subsequent sections.

Overall, no clear correlation was observed between AFM_1_ contamination in milk and the occurrence of STC, nor of AFL, while taking into account the experimental limitations highlighted, particularly for STC.

#### 2.2.1. Distribution of Mycotoxins During Cheesemaking

The distribution of mycotoxins across dairy products, processing intermediates, and by-products is influenced by multiple sources of variability. The primary source is the cheesemaking process itself, which involves milk batches of variable composition, while a secondary source is the analytical uncertainty of the quantification method. Therefore, alongside the calculation of mean distribution values, the associated uncertainty was also assessed. Results obtained from naturally contaminated milk were analyzed separately from those of the spiked milk, as AFM_1_ binds to caseins in milk and its behavior may differ from that observed in spiked milk. Spiked batches also provided a preliminary indication of STC and AFL fate. The first step involved calculating the absolute amount of each mycotoxin in the products by multiplying the measured concentration (ng/kg) by the total weight (kg). The absolute amounts are reported in the Supplementary Materials (Tables S2 and S3).

In the second step, the data obtained were normalized by setting the initial absolute amount of each mycotoxin in the starting milk to 100%, thereby allowing comparison among different batches and assessment of mycotoxin distribution across the various processing products. This distribution was assessed by calculating the Transfer Rate (%TR) according to the following equation:

(1) $${\left(\%TR\;of\;Mycotoxin\right)}_{product}\;=\;\frac{{\left(Abs.\;amount\;Mycotoxin\right)}_{product}}{{\left(Abs.\;amount\;Mycotoxin\right)}_{milk}}*100\%$$

An initial inspection of the complete dataset from the 14 experimental batches, based on the AFM_1_% transfer rate, highlighted an apparent anomaly in Batch 7 that had not been identified during the experimental phase. The remaining batches showed a consistent distribution pattern. Consequently, a formal statistical assessment was undertaken to verify whether Batch 7 qualified as an outlier.

Exploratory analysis utilized normal Q–Q plots to visually examine data distribution and detect potential extreme values. Normality was formally evaluated using the Shapiro–Wilk test (α = 0.05), while Grubbs’ test and the robust MAD-based modified Z-score served as complementary tools for outlier assessment.

Outlier identification was based on the combined statistical evidence. Following the exclusion of Batch 7, normality was reassessed using the Shapiro–Wilk test. Normality was not rejected for any of the matrices after exclusion.

The comprehensive statistical findings are presented in Tables S4 and S5.

The limited number of observations available for the statistical assessment does not allow the anomaly observed for Batch 7 to be conclusively confirmed, nor does it allow its origin to be clearly attributed to either the cheesemaking process or the analytical phase. However, the marked discrepancy observed in the data, supported by the statistical assessment, justified the exclusion of Batch 7 from all subsequent analyses.

Spiked batches were analyzed separately to investigate potential differences in AFM1 distribution relative to naturally contaminated milk. Table 3 summarizes the statistical analysis of %TR in selected batches of milk naturally contaminated with AFM_1_, including mean ± standard error of the mean (SEM), min–max values, standard deviation (SD), relative standard deviation (RSD%) and relative standard error of the mean (SEM%). Although direct measurements of AFM_1_ concentrations and weights in the curd were not available for the reasons discussed above, values were estimated either by subtracting the %TR in whey from that in milk or by summing the %TR in mozzarella and spinning water, accounting for potential AFM_1_ losses during curd processing. Table 4 presents the mass balances of AFM_1_ between the starting milk and all final products, as well as the distribution among whey, second whey, and ricotta.

Analysis of the total mass balance (milk versus ricotta, mozzarella, second whey, and spinning waters) indicated a %TR of AFM_1_ of 94.9 ± 5.9% (mean ± SEM) in the final products.

**Figure 1:**
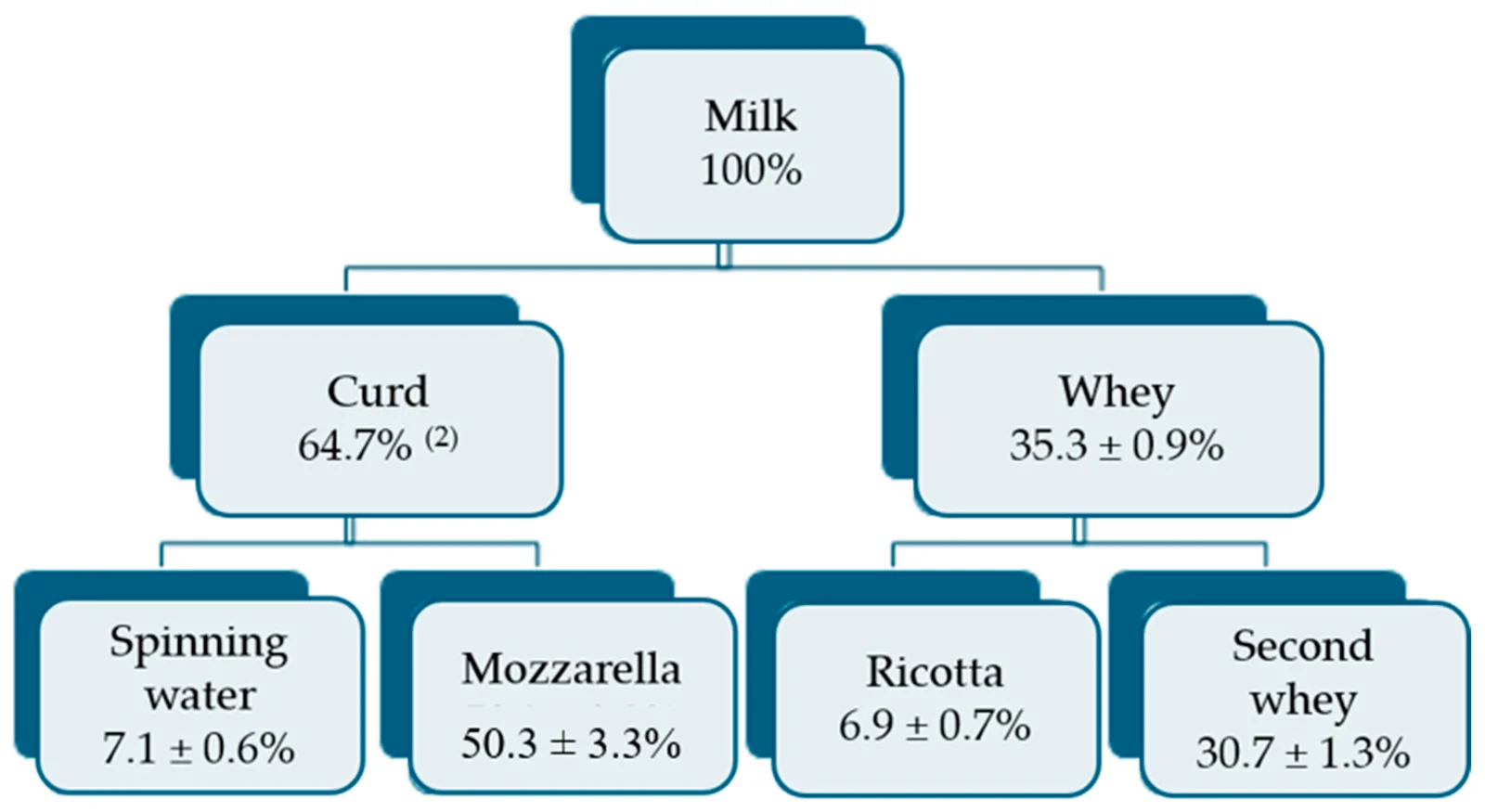
Schematic block diagram illustrating the mass balance and distribution of AFM_1_ across the different cheese processing streams and final products. Data are expressed as mean percentage ± standard error of the mean (SEM) relative to the initial AFM_1_ content in milk (100%). ^(2)^ AFM_1_ content in the curd was calculated by difference as the subtraction of the percentage found in the whey from the initial milk content (100%), yielding a value of 64.7%.

Although the mean value suggested a slight loss of AFM_1_ during cheesemaking, a one-sample Student’s *t*-test against the theoretical value of 100% (complete mass balance assumption) at *p* = 0.05 (t = −0.86, df = 12, *p* > 0.05) showed no significant deviation, with the observed %TR of AFM_1_ (94.9 ± 5.9%) therefore not significantly different from the theoretical value of 100%, indicating no evidence of AFM_1_ loss during cheesemaking within the limits of experimental uncertainty. Although the mean value suggested a slight loss of AFM_1_ during cheesemaking, a one-tailed Student’s *t*-test at *p* = 0.05 showed no significant differences. Similarly, during ricotta production, a comparison of the %TR in whey (35.3 ± 0.9%) with the sum of ricotta and second whey (%TR = 37.5 ± 2.0%) revealed no significant differences. Regarding the curd, approximate %TR values calculated using the two estimation methods were consistent, yielding 57.4% and 64.7%. Focusing on Table 3, slightly more than one-third of AFM_1_ (35.3 ± 0.9%) remained in the whey during curd formation. During ricotta production, only a small fraction of AFM_1_ (6.9 ± 0.7%) was retained in the final product while most (30.7 ± 1.3%) was lost in the second whey, corresponding to nearly 90% of the AFM_1_ present in the whey used for ricotta production. During curd processing, only a small fraction of AFM_1_ was lost in the spinning water (7.1 ± 0.6%), whereas approximately half of the initial AFM_1_ was retained in the mozzarella (50.3 ± 3.3%) (Figure 1).

The distribution of AFM_1_, STC, and AFL in the spiked batch was investigated using the same approach as before. This analysis was conducted separately, as the spiked mycotoxins may exhibit different binding affinities to caseins and, consequently, different partitioning during cheesemaking. The absolute amounts of the three mycotoxins in the different products are reported in Table S2. Table 5 shows their distribution across the various dairy products, expressed as normalized values (milk = 100%).

To facilitate a direct comparison of STC and AFL distribution relative to AFM_1_, reference ranges are included in the table. These ranges, reported as the mean ± SD of batches 1–14 (excluding batch 7, as previously stated), were adopted as a guide, rather than as mean ± SEM as used for the other data, because only a single cheesemaking trial was performed for the spiked batch. The SD values are reported in Table 3.

Table 6 reports the mass balance of the mycotoxins across different dairy products. AFM_1_ exhibited a distribution fully consistent with the reference values obtained from naturally contaminated milk, as confirmed by the mass balance.

For STC, several anomalies were previously observed in the analytical concentrations: (1) in batches 1–8, STC was detected in ricotta and mozzarella but not in the starting milk, and (2) the spiked concentrations in the milk did not match the experimentally measured values. These trends are consistent with the present findings, although the conclusions should be interpreted considering the inherent limitations of a single cheesemaking trial. As detailed in Table 5, marked deviations from reference ranges for ricotta, curd, and spinning water, which consistently exhibited higher values. The mass balance analysis demonstrates that these variations do not stem from intrinsic molecular differences relative to AFM_1_. Instead, the unusually high recoveries observed in the curd and whey fractions (122.3% relative to milk) may point to STC degradation occurring within the milk matrix.

To verify the mass balance, the actual milk spiking concentration of 50 ng/kg of AFM_1_ was factored into the calculations. As shown in Table 7, the resulting values aligned with more consistent and predictable trends. The behavior of STC data suggests a lower stability compared to AFM_1_, with degradation primarily observed in milk, although its occurrence in other products—particularly those with a high-water content (e.g., whey, second whey)—cannot be ruled out. Possibly, STC may partially degrade during the refrigerated transport to the analytical laboratory, limiting the reliability of the STC distribution data. The fate of STC in milk and during the cheesemaking processes should be further investigated in dedicated stability experiments.

AFL showed no inconsistencies between the spiked concentration in milk and the experimentally determined concentration, which may indicate greater stability of this molecule compared to STC. However, as indicated in Table 6, AFL exhibits a distinct distribution pattern compared to AFM_1_, particularly within the ricotta, curd, and mozzarella. The mass balance analysis suggests a loss of AFL during the early stages of curd formation, with an overall recovery rate of approximately 59%. However, given that AFL is a relatively poorly studied mycotoxin, similar to STC, matrix-dependent degradation cannot be excluded.

The results of the spiked milk trial, notwithstanding the limitations inherent to a single cheesemaking experiment, provide valuable evidence that both STC and AFL can transfer into dairy products during the cheesemaking process. However, further investigations are necessary to generate more robust data on their transfer rates.

#### 2.2.2. Study of Enrichment Factors (EFs) in Mozzarella and Ricotta

The EF analysis focused on AFM_1_ from cheesemaking trials conducted with naturally contaminated milk, using all available batches from 1 to 14, excluding batch 7, as well as the spiked batch. Table 8 details the EF values of AFM_1_ in mozzarella and ricotta for each batch, calculated by dividing the mycotoxin concentration measured in each product by that measured in the milk, and the statistical analysis of the data.

As expected, the highest EFs were observed in mozzarella samples with higher protein content. However, the relationship was not strictly linear, likely due to the influence of multiple contributing factors.

Across all available data, a moderate range of EF variability was observed, from 1.4 to 2.9 (RSD = 21%) for mozzarella and from 0.6 to 1.7 (RSD = 32%) for ricotta.

The normality of the enrichment factor data was assessed using the Shapiro–Wilk test, which indicated no significant departure from normality. Therefore, 95% confidence intervals (CIs) for the mean enrichment factors were calculated using the appropriate critical values from Student’s t distribution. The mean EF values were 2.3 ± 0.3 for mozzarella and 1.1 ± 0.2 for ricotta.

Even when considering the maximum EF value obtained for mozzarella (2.9), the results remain below the concentration factors reported by the Italian Ministry of Health, which applies a standard factor of 3 for soft cheeses and whey-based products [8].

This value is consistent with the available literature for soft cheese [17]; regarding ricotta, lower EFs were detailed by Cattaneo et al. (1.22–1.45) in accordance with the findings of this work [18].

The experimental EFs for milk artificially contaminated with AFM_1_, STC, and AFL are likewise reported in Table 8.

**AFM_1_ Behavior:** Regarding AFM_1_, the EF observed in mozzarella (2.9) lies at the upper end of the range recorded for naturally contaminated milk during the processing trials. This baseline was used as a reference, given that the current result derives from a single cheesemaking experiment. This outcome is somewhat unexpected, as AFM_1_ typically binds to milk caseins and is incorporated into the curd—a process generally less pronounced in artificially spiked milk. The data suggests that the 30 min homogenization following spiking may have enhanced AFM_1_-casein interactions, thereby approximating the distribution observed in naturally contaminated milk. The EF calculated for ricotta (0.9) is lower than the mean value observed in the other batches but falls well within the range of variability.

**STC behavior:** The EF values were determined from a single trial designed as a purely exploratory experiment; therefore, the interpretation of these results is subject to the inherent limitations associated with drawing definitive conclusions from a single observation. A clear discrepancy was observed between the analytically measured concentration and the nominal spiked concentration (33 ng/kg versus 50 ng/kg). Similar inconsistencies were also observed in different batches of naturally contaminated milk, where STC was detected in the final products (mozzarella and ricotta), but not in the corresponding milk samples, intermediate fractions, or by-products, suggesting possible degradation of this toxin during transport. For this reason, Table 8 reports both the experimentally determined EF values and the estimated values calculated assuming a milk concentration equal to the nominal spiking level (50 ng/kg). The EF values calculated from the experimentally measured concentrations were particularly high, especially for ricotta (EF = 2.1), whereas assuming a milk concentration of 50 ng/kg resulted in EF values falling within the range of variability observed for AFM_1_.

**AFL Behavior:** The concentrations measured in milk were consistent with the spiked levels, indicating that the mycotoxin remained stable during transport. Nevertheless, the mass balance data reported in the previous sections suggested possible degradation during experimental cheesemaking, particularly in the initial phase of curd formation. As shown in Table 8, this influenced the EF values, resulting in lower levels than those observed for AFM_1_, both in mozzarella (1.5) and particularly in ricotta (0.4).

The results obtained for the spiked batch clearly demonstrate the transfer of STC and AFL from milk to the derived products; however, further stability studies are required for both mycotoxins, and additional cheesemaking trials are necessary to obtain a more robust estimate of the enrichment factors.

## 3. Materials and Methods

### 3.1. Experimental Cheesemaking Trials with Mycotoxin-Contaminated Buffalo Milk

To evaluate the distribution and fate of AFM_1_, as well as the behavior of AFL and STC during dairy processing, laboratory-scale cheesemaking trials were conducted for mozzarella and ricotta production using:

(a) Bulk tank buffalo milk naturally contaminated with AFM_1_.
(b) Toxin-free milk artificially spiked with the three target mycotoxins (AFM1, STC, and AFL).

**Figure 2:**
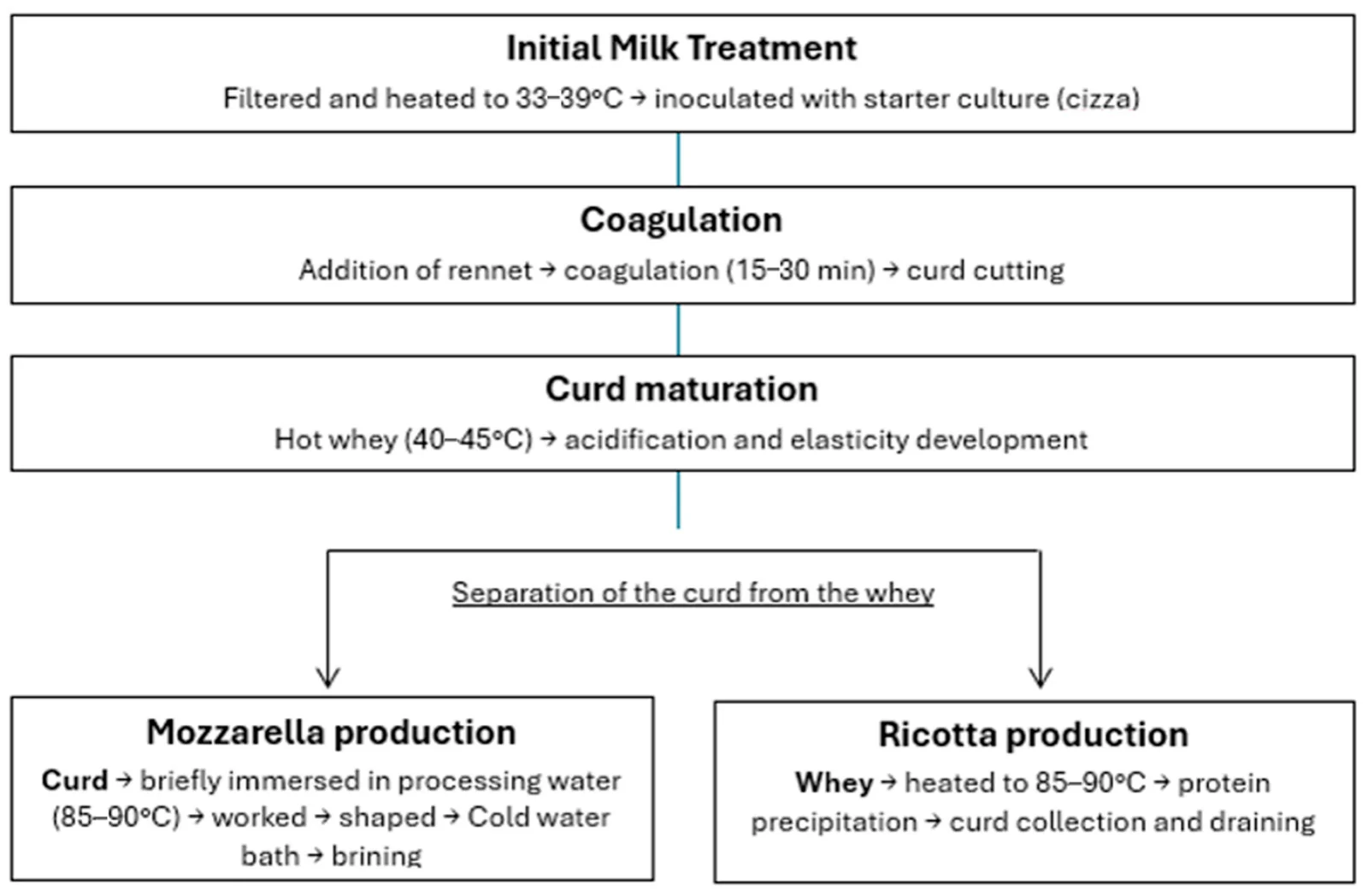
Block diagram of the Traditional Cheesemaking Method for Mozzarella and Ricotta Using Buffalo Milk.

The trials were conducted by the Istituto Zooprofilattico Sperimentale del Lazio e della Toscana “M. Aleandri” (IZSLT), which also serves as the National Reference Centre for Ovine and Caprine Milk and Dairy Products. Cheesemaking was performed under identical experimental conditions using traditional methods. A block diagram of the mozzarella and ricotta production process from buffalo milk is presented in Figure 2.

### 3.2. Experimental Cheesemaking Trials

Between July 2022 and May 2023, during routine self-monitoring activities carried out in accordance with Regulation (EC) No 853/2004, samples of bulk buffalo milk from various dairy farms located in the Lazio region were submitted to the IZSLT lab for the assessment of hygiene, health, and quality parameters [19]. Among these, fourteen samples showed AFM_1_ levels ranging from the limit of detection (LOD) to values exceeding the regulatory limit; subsequently, a specific quantity of milk required for experimental cheesemaking was collected from these farms. All milk samples were stored frozen at −20 °C until processing.

Approximately 4 kg of milk from each sample was used in separate small-scale cheesemaking trials, following traditional methods for mozzarella and ricotta production. The trials were designated as Batch 1 through Batch 14 (B1–B14).

An additional exploratory trial was performed using analyte-free milk spiked with the three target mycotoxins (AFM_1_, STC, and AFL), referred to as the “spike batch”. The spiked sample was prepared using Certified Reference Materials at concentrations of 50 ng/kg for AFM_1_ and STC, and 500 ng/kg for AFL (with respect to the milk matrix). Higher spiking concentrations for AFL were used due to the lower instrumental sensitivity and the limited knowledge regarding their behavior during cheesemaking. The milk was homogenized for 30 min prior to processing to enhance analyte binding to caseins and to better simulate naturally contaminated milk.

To allow mass balance analysis of AFM_1_, the initial milk, one intermediate product (whey), the final products (mozzarella and ricotta), and processing by-products (second whey known in Italy as ‘scotta’, and the hot water, also known as “spinning water” used for curd stretching) were weighed throughout the cheesemaking process.

Compositional analyses of milk, ricotta, and mozzarella were performed at the Istituto Zooprofilattico Sperimentale del Lazio e della Toscana “Mariano Aleandri” (IZSLT), which is accredited by ACCREDIA (accreditation number 201).

The fat and protein contents of milk and whey were determined by Fourier-transform infrared spectroscopy (FTIR) using a MilkoScan FT6000 (FOSS, Hillerød, Denmark) equipped with a specific calibration curve for buffalo milk. Mozzarella and ricotta samples were analyzed using a FoodScan Lab (FOSS, Hillerød, Denmark) near-infrared spectrophotometer. Detailed chemical profiles and product weights are provided in the Supplementary Material.

Note: The curd was not sampled due to practical limitations related to its handling and lower homogeneity, as it still contained residual whey. These factors could have compromised sample representativeness and the integrity of the cheesemaking process.

### 3.3. Analytical Method

AFM_1_, STC, and AFL determinations were carried out as reported in [15]. Briefly, 2.00 ± 0.05 g of homogenized cheese sample (5.00 ± 0.05 g for milk, whey, second whey, and spinning water) was weighed into a polypropylene centrifuge tube and spiked with 0.2 ng/g of ^13^C_17_ AFM_1_ and ^13^C_18_ STC.

The sample was mixed using an Ultra-Turrax™ (IKA-Werke GmbH & Co., Staufen im Breisgau, Germany) homogenizer with 2 mL of water and 5 mL of an ACN:MeOH solution (85:15, *v*/*v*) until a uniform suspension was obtained. For milk, whey, second whey, and spinning water the extraction mixture consisted of just 5 mL of an ACN:MeOH solution (85:15, *v*/*v*), replacing Ultra-Turrax™ homogenization with horizontal shaking (10 min). After centrifugation (14,820× *g* for 10 min), the supernatant was collected, and the residue was re-extracted using the same procedure. Combined extracts were treated with 1.0 g of NaCl and vigorously mixed to promote phase separation.

The extracts were frozen at −18 °C for 3 h to precipitate interfering substances. Immediately after freezing, 2 mL of the organic phase was diluted with 25 mL of PBS and loaded onto an AFLATEST WBSR+ immunoaffinity column for purification. Elution was performed using 1.5 mL of ACN:MeOH (1:2, *v*/*v*), which was subsequently evaporated to dryness under a nitrogen stream. The residue was reconstituted in 0.4 mL of H_2_O:ACN (75:25, *v*/*v*) with 0.1% formic acid, filtered (0.2 µm PTFE filters), and analyzed by LC-MS/MS. Quantification of AFM_1_ and STC was performed using an isotopically labeled internal standard (ILIS) calibration curve, while AFL was quantified by external calibration. The LOD and LOQ values in milk, whey, and spinning water were: AFM_1_ (0.3–0.8 ng/kg); STC (0.1–0.4 ng/kg); AFL (0.4–2 ng/kg). In mozzarella and ricotta, the values were: AFM_1_ (0.6–2 ng/kg); STC (0.2–1 ng/kg); AFL (1–5 ng/kg). Recoveries ranged from 95% to 103%, with repeatability (RSDr) between 2.0% and 4.9%. Matrix effects were negligible due to the high level of selectivity and final extract clean-up provided using immunoaffinity columns.

### 3.4. Methodological Approach

The study was structured into three phases:

1. The preliminary phase aimed to verify the successful execution of experimental laboratory-scale cheesemaking trials using buffalo milk in the laboratory by calculating the yield percentages of mozzarella and ricotta relative to the starting milk. The consistency of these findings provides substantial validity and robustness to subsequent analyses.
2. By integrating concentration data with the total weights of the respective products, the absolute amounts of mycotoxins present in the starting milk and in the corresponding intermediates, final products, and by-products were calculated. This approach enabled a comprehensive investigation of mycotoxin distribution across the different cheesemaking stages
3. Enrichment factors (EFs) were calculated from mycotoxin concentration data in the starting milk and the corresponding mozzarella and ricotta products. The mean enrichment factor and its variability were then determined.

### 3.5. Statistical Analysis

The main descriptive statistical parameters, including Mean, Standard Deviation (SD), Relative Standard Deviation (%RSD), Standard Error of the Mean (SEM) and relative Standard Error of the Mean (%SEM) were calculated using Microsoft Excel (Microsoft Excel 2007, Microsoft Corporation, version 2501, Redmond, WA, USA).

The presence of potential outliers was assessed using Grubbs’ test, with a significance threshold set at *p* < 0.05, together with the robust MAD-based modified Z-score method as a complementary approach [20].

The Shapiro–Wilk normality test (SW) and calculation of parametric 95% confidence intervals (CIs) were performed using STATGRAPHICS Centurion (STATGRAPHICS Technologies, Inc., version 19.7.02, The Plains, VA, USA).

The SEM was calculated as:

(2) $$SEM=\frac{SD}{\surd n}$$

where n is the number of observations.

The 95% confidence intervals were calculated using the formula:

CI = mean ± t_α/2,n−1_ × SEM (3)

where t_α/2,n−1_ represents the critical value of the two-tailed Student’s t-distribution for a 95% confidence level and n−1 degrees of freedom.

## 4. Conclusions

This study successfully clarified the distribution and fate of AFM_1_ across all stages of buffalo dairy processing through a comprehensive mass balance analysis.

To the best of our knowledge, this study is the first to evaluate the fate of AFL and STC during buffalo milk cheesemaking, addressing a significant gap in current regulations, which currently establish maximum limits exclusively for AFM_1_ in raw milk. This has been possible thanks to the development of a reliable and sensible methodology for these contaminants at very low concentrations in milk, cheese, and all the cheesemaking intermediate byproducts. Indeed, the primary objective of this work was to evaluate the experimental enrichment factors (EFs) for mozzarella and ricotta cheeses, which were determined to be 2.3 and 1.1, respectively. These values were substantially lower than those currently adopted by Italian regulations for non-bovine dairy products, particularly for whey-derived matrices.

The determination of EFs for milk-derived products is essential to evaluate compliance with EU Regulations. In this regard, the preliminary data for AFL and STC reported herein may support regulatory authorities in updating current thresholds for non-bovine cheeses, thereby preventing the potential under-reporting of non-compliant samples.

Regarding the secondary objectives, AFL was never detected in naturally AFM_1_-contaminated bulk buffalo milk or during the subsequent cheesemaking process. Conversely, STC was found above LOQ (1 ng/kg) almost exclusively in cheeses produced from trace-contaminated (<LOQ) starting milk.

This behavior suggests a potential instability of these aflatoxin-like compounds within this specific matrix, a phenomenon that warrants further and dedicated investigations based on the preliminary insights supplied by the data here reported.

## Figures and Tables

**Table 1:** Concentrations (ng/kg) of AFM_1_, STC, and AFL in milk and dairy products derived from naturally contaminated raw materials.

**AFM_1_**																
	**B1**	**B2**	**B3**	**B4**	**B5**	**B6**	**B7**	**B8**	**B9**	**B10**	**B11**	**B12**	**B13**	**B14**	**Mean (ng/kg)**	**Range** **(ng/kg)**
Milk	13	6.0	37	87	22	35	35	48	30	41	58	6.6	163	40	44	(6.0–163)
Whey	6.0	3.1	19	42	10	15	26	25	15	19	24	3.7	74	21	22	(3.1–74)
Second whey	5.1	3.0	19	40	10	19	26	17	14	22	26	3.8	76	21	22	(3.0–76)
Spinning water	1.9	1.2	8.2	21	7.6	5.6	15	16	6.0	7.3	13	1.8	42	7.2	11	(1.2–42)
Mozzarella	24	15	98	233	50	70	55	137	66	71	79	18	476	105	107	(15–476)
Ricotta	10	4.2	49	92	20	40	46	80	19	28	91	10	192	45	52	(4.2–192)
**STC**																
	**B1**	**B2**	**B3**	**B4**	**B5**	**B6**	**B7**	**B8**	**B9**	**B10**	**B11**	**B12**	**B13**	**B14**	**Mean (ng/kg)**	**Range** **(ng/kg)**
Milk	det	0.8	0.4	<	0.8	0.4	<	<	1.3	0.8	<	0.4	det	0.7	(0.7)	(<−1.3)
Whey	<	<	<	<	<	<	<	<	det	det	<	det	<	0.4	(0.4)	(<−0.4)
Second whey	det	<	<	<	<	<	<	<	det	det	<	det	det	det	(-)	(<−0.2)
Spinning water	<	<	<	<	<	1.7	<	<	0.4	0.8	<	det	<	det	1.0	(<−1.7)
Mozzarella	det	3.1	<	10	3.5	1.4	2.3	<	3.7	1.8	<	1.7	1.2	2.5	(3.1)	(<−10)
Ricotta	det	1.3	4.1	4.0	1.9	1.0	<	<	1.0	1.0	<	1.0	det	1.3	(1.8)	(<−4.1)
**AFL**																
	**B1**	**B2**	**B3**	**B4**	**B5**	**B6**	**B7**	**B8**	**B9**	**B10**	**B11**	**B12**	**B13**	**B14**	**Mean (ng/kg)**	**Range** **(ng/kg)**
Milk	<	<	<	<	<	<	<	<	<	<	<	<	<	<	-	-
Whey	<	<	<	<	<	<	<	<	<	<	<	<	<	<	-	-
Second whey	<	<	<	<	<	<	<	<	<	<	<	<	<	<	-	-
Spinning water	<	<	<	<	<	<	<	<	<	<	<	<	<	<	-	-
Mozzarella	<	<	<	<	<	<	<	<	<	<	<	<	<	<	-	-
Ricotta	<	<	<	<	<	<	<	<	<	<	<	<	<	<	-	-

(1) ‘det’ = detected but below the limit of quantification (LOQ). ‘<’ = below the Limit of Detection (LOD). (2) Mozzarella, Ricotta (LOD–LOQ): AFM_1_ (0.6–2 ng/kg); STC (0.2–1 ng/kg); AFL (1–5 ng/kg). (3) Milk, Whey, Second whey, Spinning water (LOD–LOQ): AFM_1_ (0.3–0.8 ng/kg); STC (0.1–0.4 ng/kg); AFL (0.4–2 ng/kg).

**Table 2:** AFM_1_, STC, and AFL: spiked concentrations (ng/kg) in milk and measured levels (ng/kg) in milk and dairy products. Recovery rates for milk in parentheses.

	AFM_1_	STC	AFL
Spiked Concentration in Milk (ng/kg)	50	50	500
Milk	51 (102%)	33 (66%)	471 (94%)
Whey	24	19	181
Second whey	24	17	177
Spinning water	16	19	158
Mozzarella	149	88	697
Ricotta	45	68	211

**Table 3:** Statistical analysis of %TR across batches (mean ± SEM, min–max, SD, RSD%, SEM%).

	Mean ± SEM (%)	Range (%)	SD	% RSD	% SEM
Milk	100	-	-	-	-
Whey	35.3 ± 0.9	30.3–39.9	3.3	9.3	2.6
Second whey	30.7 ± 1.3	22.0–37.2	4.6	14.8	4.1
Ricotta	6.9 ± 0.7	3.9–12.2	2.7	39.3	10.9
Curd	57.4 ^(1)^–64.7 ^(2)^	-	-	-	-
Spinning water	7.1 ± 0.6	3.3–12.1	2.3	32.6	9.0
Mozzarella	50.3 ± 3.3	29.3–65.5	11.9	23.6	6.6

^(1)^ Sum of the AFM_1_%TR in spinning water and mozzarella. ^(2)^ Difference between the AFM_1_%TR in milk and whey.

**Table 4:** Mass balance of AFM_1_ across the different stages of cheesemaking: Transfer Rate (%TR) from milk and between processing steps.

	%TR from Milk	%TR Between Stages
	Mean ± SEM ^(1)^ (%SEM)	Mean ± SEM (%SEM) ^(1)^
Milk	100	
Sum of Mozzarella, Spinning water, Ricotta, and Second Whey	94.9 ± 5.9 (6.2%)	94.9 ± 5.9 (6.2%)
Whey	35.3 ± 0.9 (2.5%)	
Sum of Second whey and Ricotta	37.5 ± 2.0 (5.3%)	106.2 ± 8.3 (7.8%)
Curd	64.7 ^(2)^	
Sum of Mozzarella and Spinning water	57.4 ± 3.9 (6.8%)	Approx. 89

^(1)^ Given that %TR values are derived from the same starting milk and are therefore partially dependent, a conservative approach was applied for uncertainty propagation: absolute SEMs were summed directly for total amounts, and relative SEMs (%SEM) were summed for ratios, rather than using the root-sum-of-squares method. ^(2)^ Difference between the AFM_1_%TR in milk and whey.

**Table 5:** Batch Spike: percentage transfer rates (%TR) of AFM_1_, STC, and AFL from milk to various dairy fractions, normalized by setting the initial milk content to 100%. Reference values for AFM_1_ were obtained from batches 1–6 and 8–14.

	AFM_1_	STC	AFL	%TR Reference Range (Mean ± SD)
	%TR			
Milk	100	100	100	-
Whey	34.2	41.2	27.5	32.0–38.6
Second whey	29.0	32.2	23.5	26.1–35.3
Ricotta	6.2	14.5	3.1	4.2–9.6
Curd	73.0 ^(1)^–65.8 ^(2)^	75.6 ^(1)^–58.8 ^(2)^	42.5 ^(1)^–72.5 ^(2)^	57.4 ^(1)^–64.7 ^(2)^
Spinning water	9.2	17.3	10.1	4.8–9.4
Mozzarella	63.8	58.3	32.4	38.4–62.2

^(1)^ Sum of the AFM_1_%TR in spinning water and mozzarella. ^(2)^ Difference between the AFM_1_%TR in milk and whey.

**Table 6:** Batch Spike: Mass balance of AFM_1_, STC and AFL, reporting percentage transfer rates (%TR) from milk and between processing stages. Values are normalized against an initial mycotoxin content in milk of 100%.

	AFM_1_		STC		AFL	
	%TR from Milk	%TR Between Stages	%TR from Milk	%TR Between Stages	%TR from Milk	%TR Between Stages
Milk	100		100		100	
Sum of Mozzarella, Spinning water, Ricotta, and Second Whey	108.2	108.2%	122.3	122.3%	69.1	69.1%
	AFM_1_		STC		AFL	
Whey	34.2		41.2		27.5	
Sum of Second whey and Ricotta	35.2	102.9%	46.7	113.3%	26.6	96.7%
Curd	65.8 ^(2)^		58.8 ^(2)^		72.5 ^(2)^	
Sum of Mozzarella and Spinning water	73.0	110.9%	75.6	128.6%	42.5	Approx. 59%

^(2)^ Difference between the AFM_1_%TR in milk and whey.

**Table 7:** Batch Spike: Mass balance STC across the different stages of cheesemaking. Comparison of STC Experimental and Estimated Data.

	STC Experimental Data		STC Estimated Data	
	%TR from Milk	%TR Between Stages	%TR from Milk	%TR Between Stages
Milk	100		100	
Sum of Mozzarella, Spinning water, Ricotta, and Second Whey	122.3	122.3%	80.7	80.7%
	%TR from milk	%TR between stages	%TR from milk	%TR between stages
Whey	41.2		27.2	
Sum of Second whey and Ricotta	46.7	113.3%	30.8	113.0%
	%TR from milk	%TR between stages	%TR from milk	%TR between stages
Curd	58.8 ^(2)^		72.8 ^(2)^	
Sum of Mozzarella and Spinning water	75.6	128.6%	49.9	68.6%

^(2)^ Difference between the AFM_1_%TR in milk and whey.

**Table 8:** AFM_1_, STC and AFL concentrations in naturally contaminated and spiked milk and derived dairy products, with calculated enrichment factors (EF) and statistical analysis.

**AFM_1_**					
	**Milk**	**Mozzarella**	**Ricotta**	**Mozzarella**	**Ricotta**
	**Concentration (ng/kg)**			**EF**	
Batch 1–14 ^1^ (naturally contaminated)	6.0–163	15–476	4.0–192	1.4–2.9	0.6–1.7
Spiked batch (50 ng/kg)	51	149	45	2.9	0.9
			n	13 ^2^	13 ^2^
			Mean	2.3	1.1
			SD	0.5	0.4
			%RSD	21	32
			SEM	0.13	0.10
			95% CI (t_0.975,12_ = 2.179)	2.3 ± 0.3	1.1 ± 0.2
**STC**					
	**Milk**	**Mozzarella**	**Ricotta**	**Mozzarella**	**Ricotta**
	**Concentration (ng/kg)**			**EF**	
Experimental spiked batch (50 ng/kg)	33	88	68	2.7	2.1
Estimated spiked batch (50 ng/kg) ^3^	50	88	68	1.8	1.4
**AFL**					
	**Milk**	**Mozzarella**	**Ricotta**	**Mozzarella**	**Ricotta**
	**Concentration (ng/kg)**			**EF**	
Spiked batch (500 ng/kg)	471	697	211	1.5	0.4

^1^: excluding batch 7; ^2^: excluding spiked batch. ^3^ considering STC recovery at 100%.

## Data Availability

The original contributions presented in this study are included in the article/Supplementary Material. Further inquiries can be directed to the corresponding author.

## Supplementary Materials

The following supporting information can be downloaded at https://www.mdpi.com/article/10.3390/molecules31183316/s1. Table S1: Chemical composition of milk and cheesemaking products; Table S2: AFM_1_ partition from naturally contaminated milk into cheesemaking byproducts; Table S3: AFM_1_, STC and AFX partition from spiked milk into cheesemaking byproducts. Table S4. Statistical assessment of normality and potential outliers in the %transfer rate across the 14 experimental batches using the Shapiro–Wilk test, Grubbs’ test, and MAD-based modified Z-score. Table S5. Shapiro–Wilk test results for the %transfer rate after exclusion of Batch 7.

## Abbreviations

AFB_1_	Aflatoxin B1
AFL	Aflatoxicol
AFM_1_	Aflatoxin M1
CIs	Confidence intervals
EFs	Enrichment factors
EFSA	European Food Safety Authority
EU	European Union
LC-MS/MS	Liquid chromatography coupled to mass spectrometry
LOD	Limit of detection
LOQ	Limit of quantitation
MFFB	Moisture content on a Fat-Free Basis
PDO	Protected Designation of Origin
RSD	Relative standard deviation
SEM	Standard error of the mean
STC	Sterigmatocystin
SW	Shapiro–Wilk test
%TR	Transfer rate (%)
