# Supplementary material for: Aflatoxin M1 Enrichment in Buffalo Mozzarella and Ricotta: Case Study on Naturally Contaminated Milk and Exploratory Analysis of Sterigmatocystin and Aflatoxicol
Source: Molecules. 2026 Sep 18;31(18):3316. doi: 10.3390/molecules31183316 (PMC13609493; doi:10.3390/molecules31183316)
Supplement: Supplementary file 1 [file molecules-31-03316-s001.zip › molecules-4517479-supplementary.pdf]

Table S1. Chemical composition of milk and cheesemaking products.

**Milk**				**Mozzarella**								**Ricotta**							
	**F**	**P**	**β-C**		**TSs**	**M**	**F**	**F-DM**	**P**	**S**	**NF-Ss**		**TSs**	**M**	**F**	**F-DM**	**P**	**S**	**NF-Ss**
	%	%	%		%	%	%	%	%	%	%		%	%	%	%	%	%	%
**B1**	7.5	4.6	3.3	**B1**	37	63	22	60	14	0.7	15	**B1**	24	76	15	63	13	0.6	8.8
**B2**	7.6	4.7	3.6	**B2**	53	47	32	59	20	0.8	22	**B2**	19	81	15	80	13	0.7	3.8
**B3**	4.3	4.9	3.7	**B3**	43	57	17	40	24	0.8	26	**B3**	29	71	12	41	15	0.7	17
**B4**	4.0	5.0	3.9	**B4**	40	60	11	27	26	0.8	29	**B4**	34	66	18	54	12	0.8	16
**B5**	7.6	4.5	3.3	**B5**	40	61	24	61	13	0.7	15	**B5**	28	72	20	71	13	0.6	8.2
**B6**	6.7	4.3	3.0	**B6**	37	63	20	54	18	0.8	17	**B6**	33	67	22	66	8.1	0.6	11
**B7**	6.2	4.1	2.8	**B7**	39	61	24	61	13	0.7	15	**B7**	32	68	21	66	17	0.6	11
**B8**	7.4	4.4	3.2	**B8**	43	57	20	47	22	0.8	23	**B8**	32	68	21	66	17	0.6	11
**B9**	7.8	4.0	3.0	**B9**	45	55	21	46	16	1.8	24	**B9**	NA	NA	NA	NA	NA	NA	NA
**B10**	7.6	4.1	3.1	**B10**	50	50	27	54	21	1.4	23	**B10**	30	69	17	54	11	0.8	14
**B11**	7.8	4.9	3.7	**B11**	28	72	16	56	12	0.6	12	**B11**	26	74	11	45	11	0.6	14
**B12**	8.2	4.7	4.1	**B12**	43	57	20	47	22	0.8	23	**B12**	32	68	21	65	17	0.6	11
**B13**	NA	NA	NA	**B13**	NA	NA	NA	NA	NA	NA	NA	**B13**	NA	NA	NA	NA	NA	NA	NA
**B14**	NA	NA	NA	**B14**	NA	NA	NA	NA	NA	NA	NA	**B14**	NA	NA	NA	NA	NA	NA	NA
**Batch spike**	7.8	5.1	3.8	**Batch spike**	49	51	25	50	20	1.4	25	**Batch spike**	NA	NA	NA	NA	NA	NA	NA
**mean**	6.9	4.6	3.4	**mean**	42	58	21	51	19	0.9	21	**mean**	29	71	17	61	13	1	11
**SD**	1.4	0.4	0.4	**SD**	6.7	6.7	5.2	9.7	4.5	0.4	5.2	**SD**	4.6	4.6	3.7	11	2.8	0.1	3.8
**RSD%**	19	8.0	11	**RSD%**	16	12	24	19	24	39	25	**RSD%**	16	6.4	21	19	21	13	33
**Min**	4.0	4.0	2.8	**Min**	28	47	11	27	12	0.6	12.3	**Min**	19	66	11	41	8.1	0.6	3.8
**Max**	8.2	5.1	4.1	**Max**	53	72	32	61	26	1.8	28.9	**Max**	34	81	22	80	17	0.8	17

β-C: beta casein; F: fat; F-DM: fat in dry matter; M: moisture; NF-S: non-fat solids; P: proteins; S: salt; TSs: total solids

Table S2. AFM_1 partition from naturally contaminated milk into cheesemaking byproducts.

	B 1	B 2	B 3	B 4	B 5	B 6	B 7	B 8	B 9	B 10	B 11	B 12	B 13	B 14
	**AFM1 concentration (ng/kg)**													
**Milk**	13	6.0	37	87	22	35	35	48	30	41	58	6.6	163	40
**Whey**	6.0	3.1	19	42	10	15	26	25	15	19	24	3.7	74	21
**Second whey**	5.1	3.0	19	40	10	19	26	17	14	22	26	3.8	76	21
**Spinning water**	1.9	1.2	8.2	21	7.6	5.6	15	16	6.0	7.3	13	1.8	42	7.2
**Mozzarella**	24	15	98	233	50	70	55	137	66	71	79	18	476	105
**Ricotta**	10	4.2	49	92	20	40	46	80	19	28	91	10	192	45
	**B 1**	**B 2**	**B 3**	**B 4**	**B 5**	**B 6**	**B 7**	**B 8**	**B 9**	**B 10**	**B 11**	**B 12**	**B 13**	**B 14**
	**Weight (kg)**													
**Milk**	4.02	4.01	3.98	4.03	4.01	3.97	4.00	4.02	4.01	4.02	4.09	4.03	4.01	3.98
**Whey**	2.82	2.80	3.03	3.06	2.80	2.84	3.06	2.84	2.95	3.00	2.97	2.85	3.12	3.08
**Second whey**	2.41	2.36	2.70	2.75	2.45	2.44	2.76	2.50	2.54	2.57	2.49	2.45	2.85	2.80
**Spinning water**	0.90	0.94	1.30	1.26	1.00	1.38	1.46	1.46	1.33	1.61	1.60	1.33	1.01	1.05

	B 1	B 2	B 3	B 4	B 5	B 6	B 7	B 8	B 9	B 10	B 11	B 12	B 13	B 14
**Mozzarella**	0.94	0.96	0.69	0.72	0.95	0.88	0.69	0.92	0.81	0.76	0.88	0.92	0.88	0.92
**Ricotta**	0.30	0.33	0.24	0.21	0.25	0.25	0.19	0.25	0.26	0.33	0.32	0.28	0.15	0.14
	**B 1**	**B 2**	**B 3**	**B 4**	**B 5**	**B 6**	**B 7**	**B 8**	**B 9**	**B 10**	**B 11**	**B 12**	**B 13**	**B 14**
	**AFM1 absolute levels (ng)**													
**Milk**	52	24	147	351	88	139	140	193	122	165	238	27	653	159
**Whey**	17	8.7	58	129	28	43	80	71	44	56	72	11	232	63
**Second whey**	12	7.1	51	110	24	46	72	43	37	55	65	9.3	218	59
**Spinning water**	1.7	1.1	11	26	7.6	7.7	22	23.	8.0	12	21	2.4	42	7.6
**Mozzarella**	23	14	68	168	48	62	38	126	53	54	70	17	419	97
**Ricotta**	3.0	1.4	12	19	5.1	10	8.6	20	5.0	9.0	29	2.8	29	6.2
	**B 1**	**B 2**	**B 3**	**B 4**	**B 5**	**B 6**	**B 7***	**B 8**	**B 9**	**B 10**	**B 11**	**B 12**	**B 13**	**B 14**
	**Normalization (%)**													
**Milk**	100	100	100	100	100	100		100	100	100	100	100	100	100
**Whey**	32.4	36.1	39.1	36.7	31.7	30.7		36.8	36.3	34.1	30.3	39.6	35.5	39.9
**Second whey**	23.5	29.4	34.8	31.4	27.7	33.4		22.0	30.0	33.4	27.5	35.0	33.4	37.2
**Spinning water**	3.3	4.7	7.2	7.5	8.6	5.6		12.1	6.5	7.1	8.9	9.0	6.5	4.8
**Mozzarella**	43.4	60.1	46.1	47.8	54.0	44.3		65.5	43.6	32.6	29.3	62.3	64.2	60.9
**Ricotta**	5.8	5.7	8.0	5.4	5.8	7.3		10.5	4.1	5.5	12.2	10.5	4.5	3.9

Batch 7 concentrations were outliers and not considered for further evaluations

Table S3. AFM_1, STC and AFX partition from spiked milk into cheesemaking byproducts.

AFM1 concentration (ng/kg)			
	AFM1	STC	AFL
Spiked concentration in milk (ng/kg)	50	50	500
Milk	51	33	471
Whey	24	19	181
Second whey	24	17	177
Spinning water	16	19	158
Mozzarella	149	88	697
Ricotta	45	68	211
**Weight (kg)**			
Milk	4.02		

Whey	2.88		
Second whey	2.51		
Spinning water	1.21		
Mozzarella	0.88		
Ricotta	0.28		
**AFM1 absolute levels (ng)**			
	AFM1	STC	AFL
Milk	205	133	1893
Whey	70	55	521
Second whey	60	43	444
Spinning water	19	23	191
Mozzarella	131	77	613
Ricotta	13	19	60
**Normalization (%)**			
	AFM1	STC	AFL
Milk	100.0	100.0	100.0
Whey	34.3	41.2	27.5
Second whey	29.1	32.2	23.5
Processing water	9.3	17.3	10.1
Mozzarella	63.9	58.3	32.4
Ricotta	6.2	14.5	3.1

Table S4. Statistical assessment of normality and potential outliers in the %transfer rate across the 14 experimental batches using the Shapiro–Wilk test, Grubbs' test, and MAD-based modified Z-score.

**Matrix**	**n**	**Shapiro–Wilk W**	**p-value**	**Normality**	**Grubbs' G**	**p-value**	**Modified Z-score (MAD)**	**Potential outlier**	**Batch**
Whey	14	0.757	0.002	Rejected	3.046	<0.001	4.411	56.8	7

Second whey	14	0.883	0.064	Not rejected	2.722	0.013	4.553	51.3	7
Spinning water	14	0.907	0.144	Not rejected	2.503	0.051	3,454	—	—
Mozzarella	14	0.921	0.227	Not rejected	1.653	1.000	—	—	—
Ricotta	14	0.870	0.042	Rejected	2.090	0.320	---	—	—

Table S5. Shapiro–Wilk test results for the %transfer rate after exclusion of Batch 7

**Matrix**	**n**	**Shapiro–Wilk W**	**p-value**	**Normality**
Whey	13	0.933	0.372	Not rejected
Second whey	13	0.944	0.510	Not rejected
Spinning water	13	0.968	0.877	Not rejected
Mozzarella	13	0.923	0.278	Not rejected
Ricotta	13	0.873	0.058	Not rejected
